# First detection of *Candidatus* Rickettsia tarasevichiae in *Hyalomma marginatum* ticks

**DOI:** 10.1371/journal.pone.0296757

**Published:** 2024-02-02

**Authors:** Si Su, Meng-Yu Cui, Zheng Gui, Qi-Qi Guo, Hong Ren, Shi-Fa Ma, Lan Mu, Jing-Feng Yu, Shao-Yin Fu, Dong-Dong Qi

**Affiliations:** 1 Graduate School, Inner Mongolia Medical University, Hohhot, Inner Mongolia, China; 2 First Hospital of Jilin University, Changchun, China; 3 First Clinical College, Inner Mongolia Medical University, Hohhot, Inner Mongolia, China; 4 Laboratory of Basic and Clinical Psychiatry, The Third People’s Hospital of Hulunbuir City, Hulunbuir, Inner Mongolia, China; 5 School of Basic Medicine, Inner Mongolia Medical University, Hohhot Inner Mongolia, China; 6 Inner Mongolia Academy of Agricultural & Animal Husbandry Science, Hohhot, Inner Mongolia, China; University of Bari, ITALY

## Abstract

Ticks are important vectors of zoonotic diseases and play a major role in the circulation and transmission of many rickettsial species. The aim of this study was to investigate the carriage of *Candidatus* Rickettsia tarasevichiae (CRT) in a total of 1168 ticks collected in Inner Mongolia to elucidate the potential public health risk of this pathogen, provide a basis for infectious disease prevention, control and prediction and contribute diagnostic ideas for clinical diseases that present with fever in populations exposed to ticks. A total of four tick species, *Haemaphysalis concinna* (n = 21), *Dermacentor nuttalli* (n = 122), *Hyalomma marginatum* (n = 148), and *Ixodes persulcatus* (n = 877), were collected at nine sampling sites in Inner Mongolia, China, and identified by morphological and molecular biological methods. Reverse transcription PCR targeting the 16S ribosomal RNA (*rrs*), *gltA*, *groEL*, *ompB* and *Sca4* genes was used to detect CRT DNA. Sequencing was used for pathogen species confirmation. The molecular epidemiological analysis showed that three species of ticks were infected with CRT, and the overall positive rate was as high as 42%. The positive rate of *I*. *persulcatus* collected in Hinggan League city was up to 96%, and that of *I*. *persulcatus* collected in Hulun Buir city was 50%. The pool positive rates of *D*. *nuttalli* and *H*. *marginatum* collected in Bayan Nur city and *H*. *concinna* collected in Hulun Buir city were 0%, 28% and 40%, respectively. This study revealed the high prevalence of CRT infection in ticks from Inner Mongolia and the first confirmation of CRT detected in *H*. *marginatum* in China. The wide host range and high infection rate in Inner Mongolia may dramatically increase the exposure of CRT to humans and other vertebrates. The role of *H*. *marginatum* in the transmission of rickettsiosis and its potential risk to public health should be further considered.

## Introduction

*Rickettsia* species are a group of important, obligate intracellular, vector-borne pathogens that cause rickettsioses through arthropod vectors such as fleas, ticks, mites or lice [1–3]. Rickettsial diseases constitute a significant public health concern and impose a substantial economic burden on a global scale [3]. *Candidatus* Rickettsia tarasevichiae (CRT), an emerging tick-borne human pathogen, was initially detected in *Ixodes persulcatus* collected from various regions of Russia, including western Siberia, eastern Siberia, and the southern Urals in 2003 [4]. Since then, it has been detected in *I*. *persulcatus*, *Haemaphysalis japonica douglasi*, *Dermacentor silvarum*, *Ixodes pavlovskyi*, *Ixodes trianguliceps* and *Haemaphysalis concinna* within the territory of Russia [5–9]. The presence of CRT has been further documented in *I*. *persulcatus* in Estonia [10], Japan [11, 12], Mongolia [13], Korea [14] and the Chinese-Russian border [6]. It has also been detected in rodents and ticks in the northeastern region of China [15–18]. Few cases have been reported in Inner Mongolia, probably due to misdiagnosis resulting from a lack of understanding of CRT. This means that there is a wide range of invertebrate hosts infected with CRT, which threatens human health in the context of increased human activity in wildlife habitats. Despite the limited reports on CRT in Inner Mongolia [19, 20], this region is of great importance due to its vast land area, ecological and environmental diversity, and rich tick species reserve. Inner Mongolia encompasses three distinct ecological zones, including forest, grassland, and Gobi and semi desertification steppe areas from east to west, which makes it a unique and complex environment for tick-borne diseases. Furthermore, the region spans three climatic zones, from east to west, including temperate semi humid, semi arid, and arid zones, which also contribute to the diversity of tick species and their distribution. Therefore, further research is needed to understand the distribution and prevalence of CRT in Inner Mongolia, which could provide valuable insights into the epidemiology and control of tick-borne diseases in China.

However, recent studies have shown that CRT is pathogenic to humans and can even cause death in humans [21–23]. Human infection with CRT was first reported in northeastern China in 2012 [24]. In 2014, eight patients in eastern central China who were infected with CRT and presented with symptoms resembling severe fever with thrombocytopenia syndrome (SFTS) died as a result of misdiagnosis [22]. In May 2017, a fatal case of tick-borne rickettsiosis caused by mixed *Rickettsia sibirica* subsp. *sibirica* and CRT infection was reported in Russia [23].

Ticks are considered second to mosquitoes as vectors of human diseases in the world and can carry and transmit hundreds of pathogens, including viruses, bacteria, protozoa and helminths [25, 26]. The prevalence and biodiversity of *Rickettsia* spp. in *I*. *persulcatus*, *H*. *concinna*, *H*. *douglasi*, *Dermacentor nuttalli* and *D*. *silvarum* ticks from different regions in Inner Mongolia were examined in our previous study [19, 27, 28]. However, there is a lack of research data on CRT, and it is necessary to investigate the status of CRT infection in Inner Mongolia. To this end, we conducted screening for CRT infection in ticks collected from regions where there have been previous reports of tick-borne pathogens (TBPs) [29].

## Materials and methods

### Ethics statement

The collection of ticks from the body surface of cattle, sheep and goats in this study was verbally approved by the animals’ owners and performed in strict accordance with the National Guidelines for Experimental Animal Welfare of China (2006–398). In addition, this study was reviewed and approved by the Medical Ethics Committee of Inner Mongolia Medical University (No. YKD202302084).

### Sample collection and tick species identification

**Fig 1:**
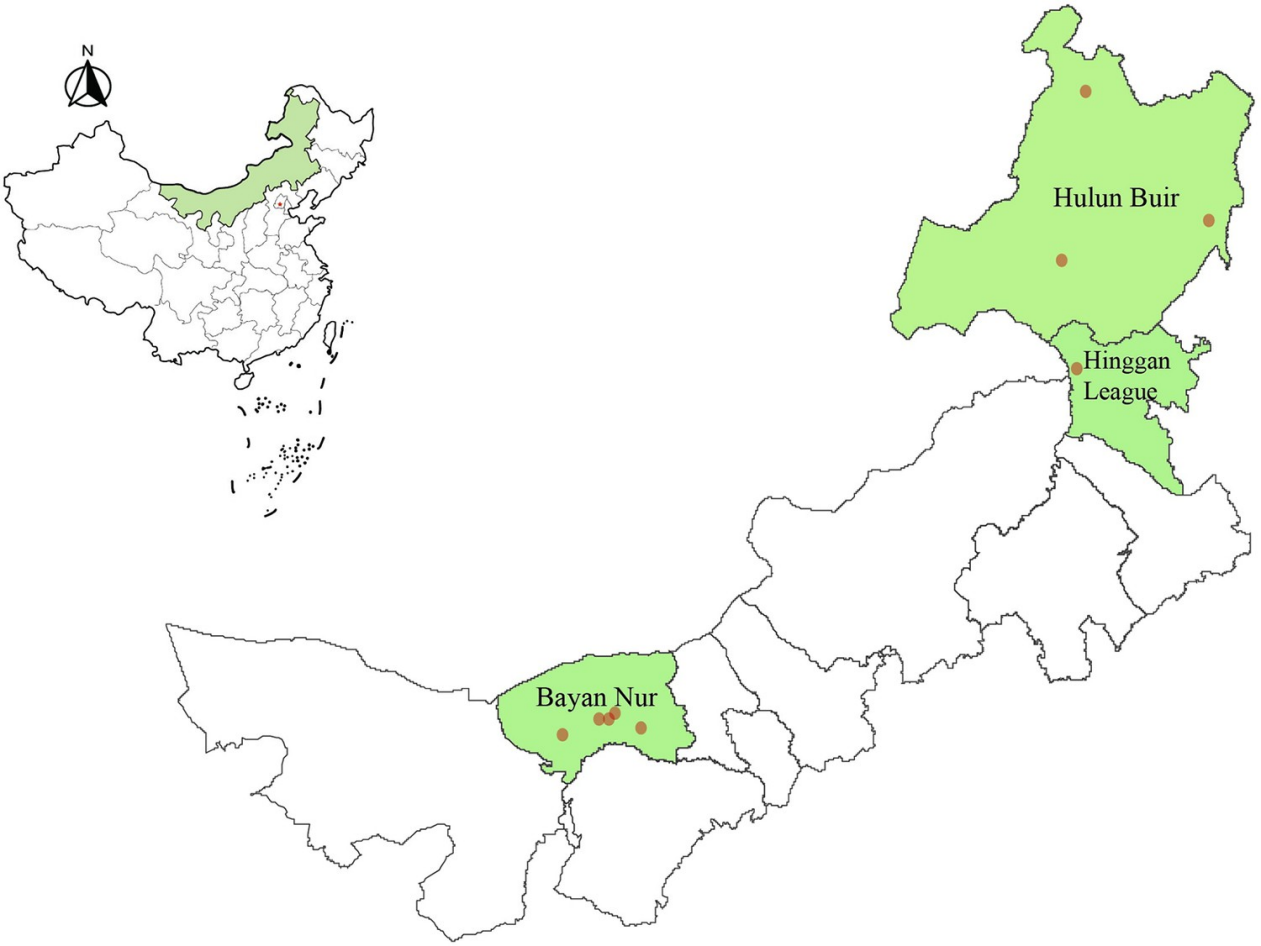
Map of tick collection sites in Inner Mongolia, China. Map is created using the Software program ArcGIS. Map source: Xinliang Xu. Multi-year administrative division boundary data of Chinese cities and municipalities. Resource and Environmental Science Data Registration and Publication System. (http://www.resdc.cn/DOI), 2023. DOI:10.12078/2023010102.

From May 2021 to May 2023, ticks were collected at nine sampling sites with relatively high tick population densities in three central cities, Hulun Buir, Hinggan League, and Bayan Nur, in Inner Mongolia, China (Fig 1 and Table 1). Ticks were collected directly from domestic animals (cattle, sheep and goats) and grasslands and then brought back to the laboratory alive. The tick species were initially identified based on their morphological characteristics, such as coxae, scutum, genital opening, anal aperture and spiracular plates, using stereomicroscopes [30–34]. The classification was further confirmed through gene sequencing of cytochrome c oxidase subunit 1 (*COI*) [35] or small subunit 16 S ribosomal RNA (*rrs*) [36]. Following morphological identification, ticks were stored at −80°C until nucleic acid extraction.

**Table 1:** Tick sampling information.

Species of ticks	Group	Animal/Geomorphy	Region	Tick (n)	Coordinate	
					Longitude	Latitude
*Ixodes persulcatus*	D	Forest shrub	HingganLeague	617	120°6’	47°3’
*Dermacentor nuttalli*	B	Goat/Sheep	BayanNur	118	109°36’	40°55’
				3	107°43’	41°7’
				1	107°59’	41°7’
*Hyalomma marginatum*	E	Goat	BayanNur	136	109°36’	40°55’
				3	107°43’	41°7’
				2	107°59’	41°7’
				1	106°24’	40°33’
				6	108°32’	41°19’
*Ixodes persulcatus *	K	Forest shrub	HulunBuir	176	121°51’	51°41’
				84	120°42’	49°17’
*Haemaphysalis concinna*	G	Cattle/Dog	HulunBuir	21	124°35’	49°45’
Total				1168		

### Nucleic acid extraction

To eliminate potential impurities and pathogens from the surface of ticks, each tick was immersed in 75% ethanol for three minutes, washed three times with phosphate-buffered saline (PBS), and finally air-dried on sterile filter paper. After being divided into 130 tick pools of 1–50 ticks depending on feeding status and collection location, the ticks were crushed in a mortar with a sterile pestle at a low temperature maintained by liquid nitrogen and placed in a 1.5 ml centrifuge tube. Total RNA was extracted from each tick pool sample using the TransZol Up Plus RNA Kit (TransGen Biotech, China), and cDNA was then obtained using TransScript® II One-Step gDNA Removal and cDNA Synthesis SuperMix (TransGen Biotech, China) according to the instructions provided by the manufacturer.

### Detection of pathogens

cDNA extracted from ticks was examined using polymerase chain reaction (PCR) targeting the *rrs* gene, and the amplified gene fragments were sequenced to detect and identify *Rickettsiales* bacteria in the ticks. For further identification of CRT, the *rrs*-positive tick samples were further amplified by PCR targeting the *gltA*, *groEL*, *ompB* and *Sca4* genes and sequenced. DNA samples were considered CRT-positive when at least two of these four genes were positive.

DNA was amplified using a system of 20 μl, including 11 μL PCR Master Mix (TianGen, China), 1 μl of DNA from each sample, and 0.5 μl each of reverse and forward primer, and filled to volume with double-distilled water. Negative and positive controls were included in each PCR. The amplified PCR products were separated on a 1.5% agarose gel and purified using the Gel DNA Recovery Kit (TianGen, China) according to the manufacturer’s instructions for sequencing. Oligo7 (https://www.oligo.net/downloads.html) was used to design specific primers. The synthesis of primers and sequencing were performed by Sangon Biotech (Shanghai, China). The primers used for amplification are listed in Table 2.

**Table 2:** Primer sequence list for *Candidatus* Rickettsia tarasevichiae detection.

Gene	Name	Primer sequence	Annealing temperature(°C)	Amplicon size(bp)
*gltA*	F	GTCGGTTCTCTTTCAGCATT	57	311
	R	CCGGCAATTCTTACTGTTGA	57	
*ompB*	F	GCAACAACTACAGGAACCAC	56	254
	R	AACTGGCTACTTCCGATAGC	56	
16S rRNA	F	TGATCCAGCAATACCGAGT	56	382
	R	TGATCCAGCAATACCGAGT	55	
*groEL*	F	AGGTCCAAAAGGAAGAAACG	56	354
	R	GTACCGACCTGTGCTATTTC	56	
*sca4*	F	CACTGCTCACTACGAAGAAG	56	358
	R	TGAAGATTCAGCTTGTTGCA	56	

### Phylogenetic analysis

The BLAST nucleotide collection database (nr/nt) was utilized to identify homologies in our gene sequences. The acquired nucleotide sequences from *Rickettsiae* target genes were edited and assembled using MEGA software version 7.0, while MAFFT v.7.266 was used for multiple nucleotide sequence alignment [37, 38]. Phylogenetic trees were constructed using the maximum likelihood (ML) approach of version 7.0 of the MEGA program, and bootstrap analysis with 1000 replicates was performed to evaluate branch reliability. Branches with values greater than 70% support were deemed significantly different for presentation. The phylogenetic trees were modified and visualized using FigTree v.1.4.3.

### Statistical analysis

The gathered data were statistically analyzed using the Statistical Package for Social Sciences Version 21.0 software (SPSS, Chicago, IL, USA). To determine the differences in the positive rate of rickettsiae, the p value was calculated using the Chi-square test or Fisher’s exact test. The statistical significance level was set at p **<** 0.05.

## Results

### Species and distribution of the collected ticks

In Inner Mongolia, 1,168 adult ticks were collected from the nine sampling regions of Hulun Buir, Hinggan League, and Bayan Nur. The ticks were all classified as one of four species belonging to four different genera based on their morphological characteristics and gene sequencing data, including *H*. *concinna* 2% (21/1168), *D*. *nuttalli* 10% (122/1168), *H*. *marginatum* 13% (148/1168), and *I*. *persulcatus* 75% (877/1168) (Fig 1 and Table 1). Based on the sample point locations, only *I*. *persulcatus* 53% (617/1168) were found in Hinggan League. *D*. *nuttalli* (10%, 122/1168) and *H*. *marginatum* (13%, 148/1168) were discovered in Bayan Nur. *I*. *persulcatus* 22% (260/1168) and *H*. *concinna* 2% (21/1168) were collected in Hulun Buir (Fig 1 and Table 1). A total of 130 tick pools were created based on region, species, and feeding status for all ticks included in the study. Sequences were entered in GenBank with accession numbers OR272248-OR272253, OR294053-OR294057, OQ852069 and OQ852073.

**Fig 2:**
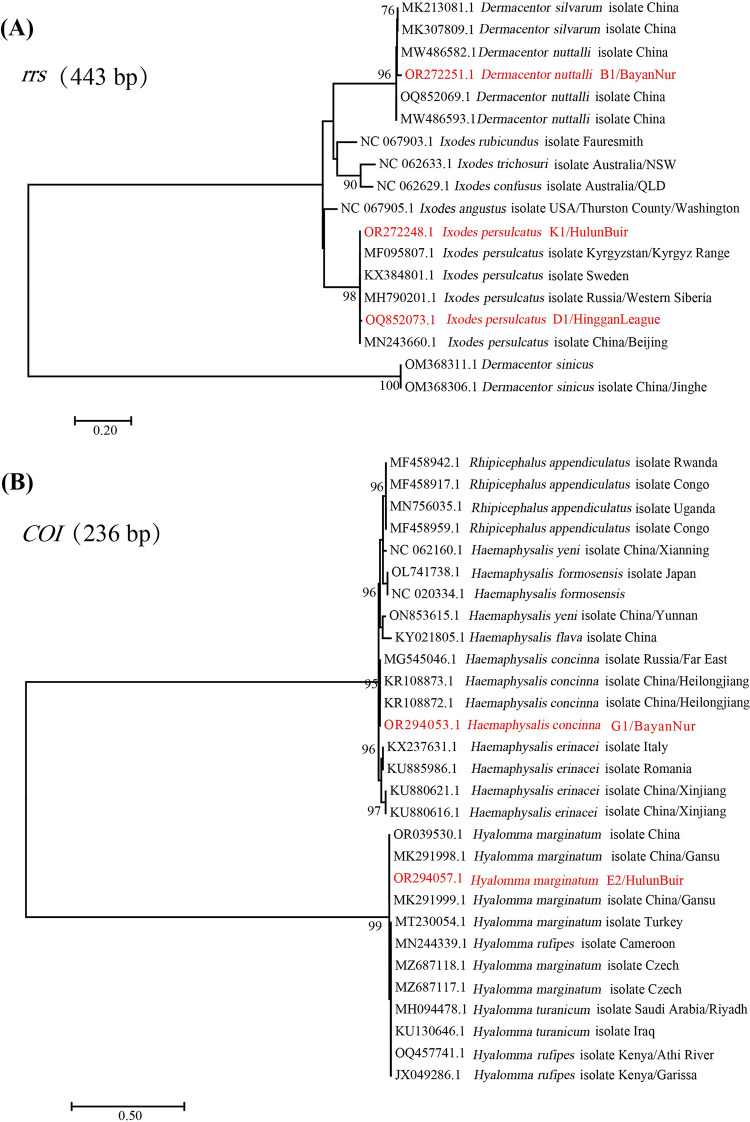
Identification of the ticks based on phylogenetic analysis with the *rrs* gene and *COI* gene. The red font indicates the representative sequences from the ticks in the different sampling regions.

The phylogenetic trees shown in Fig 2A and 2B are based on the sequences of the *rrs* and *COI* genes. The sequences acquired in the present study were clustered with their respective homologs, corresponding to the four species (Fig 2).

### *Rickettsia* bacteria detected in ticks

*rrs* gene-positive samples were utilized for the identification of CRT-specific segments of the *gltA*, *ompB*, *gloE*L, and *sca4* genes. The RT‒PCR results revealed a total positivity rate of 42% (55/130), with the highest pool positivity rate observed in the entire *I*. *persulcatus* tick population collected from Hinggan League at 96% (25/26). Additionally, the pool positivity rates for *H*. *marginatum* ticks collected from Bayan Nur and for both *I*. *persulcatus* and *H*. *concinna* ticks collected from Hulun Buir were 28% (14/50), 50% (7/14) and 40% (4/10), respectively. However, no simultaneous detection of two or more CRT-specific genes was observed in any pool of *D*. *nuttalli* ticks collected in Bayan Nur (Table 3).

**Table 3:** Prevalence of *Candidatus* Rickettsia tarasevichiae detected in tick samples collected in Inner Mongolia, China.

Parameters	NO.tested (n/ %)	The pool positivity rate (n/ %)	X^2^-value	P
**Region**				
HingganLeague	26/ 20%	25/ 96%	51.965	<0.001
BayanNur	80/ 62%	14/ 18%		
HulunBuir	24/ 18%	11/46%		
**Species of ticks**				
*Dermacentor nuttalli*	30/ 23%	0/ 0%	50.232	<0.001
*Hyalomma marginatum*	50/ 38%	14/ 28%		
*Ixodes persulcatus *	40/ 31%	32/ 80%		
*Haemaphysalis concinna*	10/ 8%	4/ 40%		
**Species / Region**				
*Ixodes persulcatus*/HingganLeague	26/ 20%	25/ 96%	58.422	<0.001
*Dermacentor nuttalli*/BayanNur	30/ 23%	0/ 0%		
*Hyalomma marginatum*/BayanNur	50/ 38%	14/ 28%		
*Ixodes persulcatus*/HulunBuir	14/ 11%	7/ 50%		
*Haemaphysalis*/concinnaHulunBuir	10/ 8%	4/ 40%		
Total(pools)	130	55/ 42%		

Analyzing the regional distribution of positive samples, there were differences in the pool positivity rates among the sampling sites. The highest positive rate was found in Hinggan League (25/26, 96%), followed by Hulun Buir (11/24, 46%) and Bayan Nur (14/80, 18%). The analysis results of the collected tick species showed that there were also differences in infection rates among the four different tick species from the genus *Ixodes*. CRT was the most widely infected and had the highest pool positivity rate among the *I*. *persulcatus* ticks (32/40, 80%), followed by *H*. *concinna* ticks (4/10, 40%) and *H*. *marginatum* ticks (14/50, 28%), and was not detected among the *D*. *nuttalli* ticks collected in the present study.

### Genetic and phylogenetic analysis

All CRT gene sequences obtained in this study have been deposited into GenBank under accessions OR454091-OR454142, OR449962-OR449989 and OR712487-OR712557. Sequence BLAST analysis showed that the nucleotide similarity between the *gltA*, *groEL*, *ompB* and *sca4* genes that we obtained and the corresponding sequences of CRT available in GenBank were 99.55%-100%, 100%, 100%, and 100%, respectively. The nucleotide similarity of all sequences obtained in this investigation to the published sequences on NCBI exceeds 99%.

The *ompB* gene sequences in this study exhibited the highest similarity with Pr-7477 (OP722685.1) from *I*. *persulcatus* in the Far East, Primorsky Krai, Russia [39], while the *gltA* and *groEL* gene sequences showed the greatest resemblance to Bayan-68 (MN450397.2, MN450404.2) from *I*. *persulcatus* in Harbin, China [40]. Furthermore, the *sca4* gene sequence of the Om-111 (OQ540735.1) isolate of *I*. *persulcatus* discovered in Western Siberia’s Omsk Province shared a high degree of similarity with the *sca4* gene sequences identified in this study.

**Fig 3:**
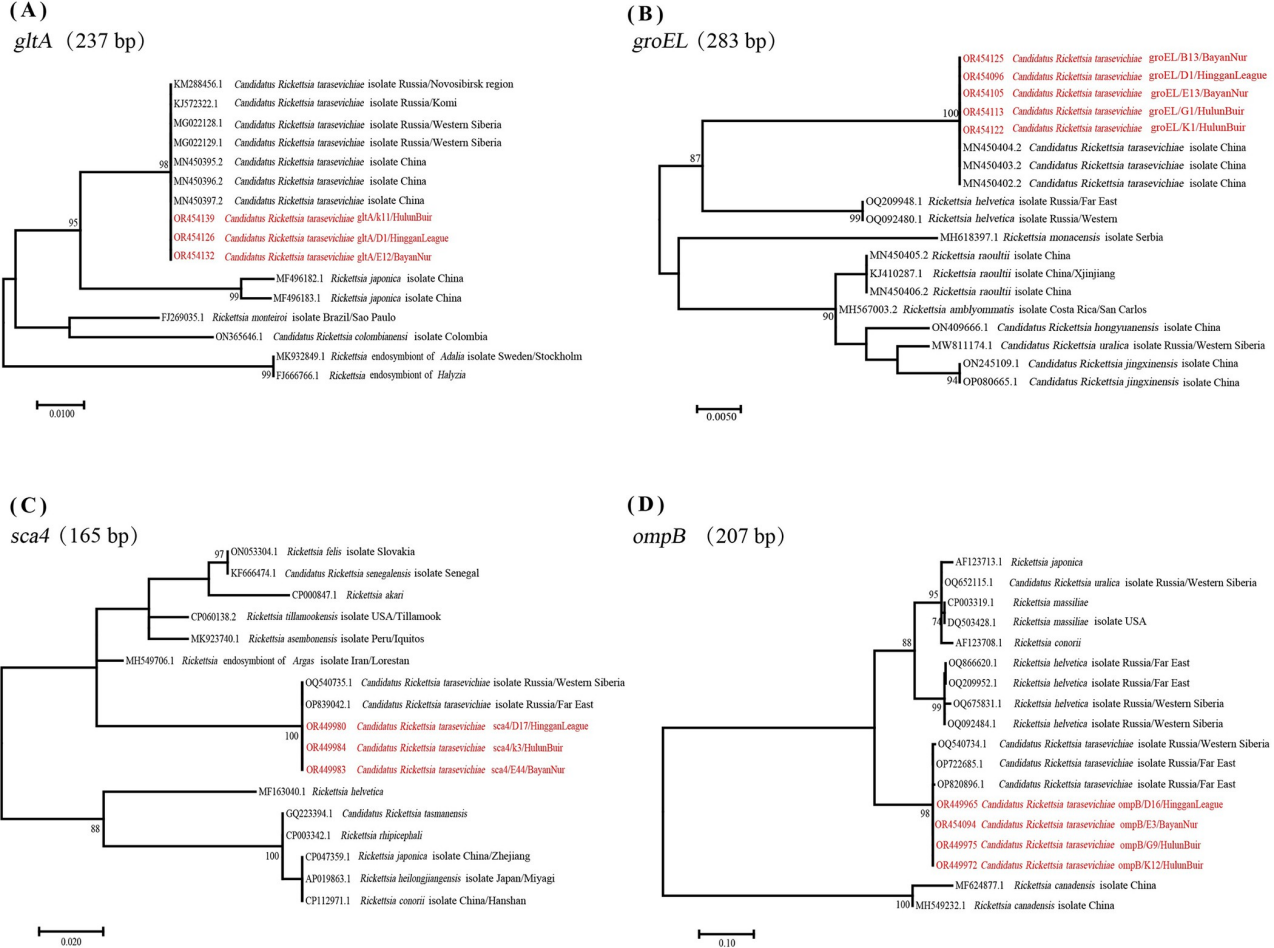
Phylogeneic tree based on the *gltA*, *groEL*, *ompB* and *sca4* genes of CRT. The genetic identity among different *Rickettsia* species was inferred by the maximum-likelihood method implemented in MEGA7 and rooted by the midpoint method. The red font indicates the nucleotide sequences of the *gltA*, *groEL*, *ompB* and *sca4* gene segments from CRT in the different sampling regions.

In the phylogenetic trees constructed in MEGA7 using maximum likelihood (ML) based on the *gltA*, *groEL*, *ompB*, and *sca4* genes, the sequences representative of this study were clustered with the corresponding published CRT sequences obtained in NCBI (Fig 3).

## Discussion

In this study, the distribution and prevalence of CRT among four tick species collected from nine sampling sites in three leagues in Inner Mongolia were investigated. A total of 1168 ticks, including *D*. *nuttalli*, *I*. *persulcatus*, *H*. *concinna*, and *H*. *marginatum*, were collected for this purpose in northern China. Inner Mongolia covers an area of 1,183,000 square kilometers, accounting for 12.3% of China’s land area. The suitable climatic conditions and geographical advantages provide advantages for the development of animal husbandry as well as a good living environment for ticks. Furthermore, the borders of Inner Mongolia with Mongolia and Russia facilitate cross-regional transmission of tick-borne pathogens. Since CRT was first identified in *I*. *persulcatus* collected in Russia [4] and subsequently found to be widely distributed in this species, many *I*. *persulcatus* ticks were collected for this study. *D*. *nuttalli*, a dominant tick species in Inner Mongolia, is known to have a wide distribution and can carry various species of *Rickettsia* [28, 29]. In a survey of microbial diversity carried by *D*. *nuttalli* ticks collected in Hulun Buir, CRT was reported to be present [20, 27]. However, there is limited research on CRT in *H*. *concinna* and *H*. *marginatum*. CRT infection has been reported in *H*. *concinna* [41], but there have been no reports of CRT carriage and transmission by *H*. *marginatum*. The Sca family is one of the largest protein families in *Rickettsia*. Certain members of the family, such as *ompB* and *sca4*, are known to be antigenic determinants of Spotted Fever Group (SFG) or Typhus Group (TG) rickettsiae [42]. The *gltA* and *groEL* genes have also been proven to be useful for the molecular identification and characterization of *Rickettsia* [43, 44]. In this study, specific primers were used to screen the *gltA*, *ompB*, *groEL*, and *sca4* genes of CRT in the samples. The presence of CRT infection was demonstrated in *H*. *marginatum* ticks collected from Bayan Nur in Inner Mongolia, marking the first detection of CRT in *H*. *marginatum*. This finding may have important implications for the prevention and control of tick-borne rickettsioses caused by CRT infection in the local population.

Different species of ticks and different sampling locations showed variations in the prevalence of CRT. Among the four tick species collected in this study, *I*. *persulcatus* had the highest infection rate of CRT (80%). Pathogens were detected in *I*. *persulcatus* samples collected from Hinggan League (96%) and Hulun Buir (46%) cities. The next highest infection rate was observed in *H*. *concinna* ticks collected from Hulun Buir (40%), followed by *H*. *marginatum* ticks collected from the Bayan Nur region (28%). No CRT infection was detected in *D*. *nuttalli* ticks collected from the same region. Previous reports have identified CRT in various tick species, including *I*. *persulcatus*, *H*. *japonica douglasi*, *D*. *silvarum*, *I*. *pavlovskyi*, *I*. *trianguliceps*, and *H*. *concinna*, within the territory of Russia [5–9]. In neighboring Mongolia, the infection rate of CRT in *I*. *persulcatus* ranged from 19.5% to 46.6% [13], which is consistent with our research findings. It has also been reported that CRT was detected in 5.42% of *D*. *nuttalli* ticks in Hulun Buir, Inner Mongolia [20]. However, we did not detect CRT infection in *D*. *nuttalli* ticks, possibly due to differences in climate and environment between the sampling sites. The Hulun Buir sampling site is located in the northwest, adjacent to Russia and Mongolia, while the southeastern region is adjacent to Heilongjiang Province. These areas share similar natural environments and habitats, which may facilitate the cross-regional transmission of the pathogen. The differences in CRT infection rates observed in *H*. *marginatum* ticks collected from the Bayan Nur region may be attributed to specific collection locations and differences in the host animals they inhabit. These results provide epidemiological data to support the prevention and control of ticks and tick-borne diseases in Inner Mongolia, China, and contribute to our understanding of the epidemiology of CRT.

The *gltA* and *groEL* genes obtained in this study showed the highest similarity to the *gltA* and *groEL* genes isolated from Bayan-68 of *I*. *persulcatus* in Harbin, China. Similarly, the *ompB* and *sca4* gene sequences were most similar to the *ompB* and *sca4* genes of CRT isolated from Russia. This similarity may be due to the proximity of Inner Mongolia to northeastern China and the Far East/Siberia region of Russia, which share similar natural environments and habitats. Additionally, there are limited genetic data available for the *ompB* and *sca4* genes of CRT in northeastern China.

CRT has been confirmed as the pathogen causing human rickettsioses and belongs to spotted fever group rickettsiae (SFGR) [45]. The first five cases of human infection with CRT were reported in northeastern China in 2012 [21]. The patients were hospitalized with symptoms such as fever, asthenia, anorexia, nausea, headache, eschar, and lymphadenopathy. Initially, their conditions were misdiagnosed because none of the patients presented with the typical rash associated with spotted fever group rickettsiae infections in China. The results of this study demonstrate the exposure of cattle, goats, and humans in Inner Mongolia to CRT. Therefore, investigating the distribution of CRT may contribute to establishing an etiological diagnosis, facilitating appropriate treatment, and implementing public health measures. This study unveils the high prevalence of CRT infection in ticks from Inner Mongolia, and it offers valuable molecular epidemiological data regarding CRT infection not only in Inner Mongolia but also in certain regions of North China, Northwest China, and Northeast China. The detection of CRT infection in *H*. *marginatum* for the first time suggests a possible etiological diagnosis for tick-borne rickettsioses caused by *H*. *marginatum* bites and contributes to the surveillance of human rickettsioses in China.

## Supporting information

S1 File (ZIP)
